# P-1006. If You Build It Will They Come?: Lessons Learned from a Dedicated Congenital Syphilis Clinic

**DOI:** 10.1093/ofid/ofae631.1196

**Published:** 2025-01-29

**Authors:** Ryan H Rochat

**Affiliations:** Baylor College of Medicine, Houston, Texas

## Abstract

**Background:**

Cases of congenital syphilis (CS) have been rising for the past decade, with 3755 infants affected in 2022 alone. Texas accounted for nearly 25% of these children, with 922 cases reported in 2022, up 35% from the year prior. A review of the management of these children across our system of over 2000 medical professionals suggested that a clinic dedicated to CS, may be warranted to ensure that these children receive evaluations as recommended by the CDC. Here we report on the logistics of this clinic with insights into its success and failures, and the implication of these findings for the general pediatric population and its providers.

**Methods:**

Laboratory and encounter specific data for children diagnosed with CS from 1/1/2010 to 12/31/2021 was obtained from our Epic® electronic health record (EHR). Additionally, we requested scheduling and appointment information for patients evaluated in our CS clinic at Texas Children’s Hospital which started in 2022.

**Results:**

Prior to the establishment of our dedicated CS clinic in 2022, we identified 458 children born into our hospital system and had been evaluated for CS from 2010-2021. Of these 458 infants, 233(51%) had a subsequent encounter within our system at ≥2months of age, with only 66(28%) of them having a repeat RPR. From 4/2022-12/2023 there were a total of 367 outpatient encounters in our CS clinic (239 completed, 128 no show/cancelled). All children seen in our CS clinic had repeat RPR testing, in addition to age-appropriate developmental screening, according to CDC recommendations.

**Conclusion:**

The rising rate of CS in the US should concern all pediatricians. Our findings call attention to the reality that follow up is not guaranteed and recommendations are not universally followed. Given that CS cases were at lifetime lows a decade ago, there is a concern that providers may be unaware of current management recommendations for these children. Integrated healthcare systems, coupled with clinical informatics, give us the opportunity to address these problems in real-time and make sure no child falls through the cracks. Our results and experiences from this unique clinic will be invaluable in providing timely feedback for tailoring education to providers and families to make sure that these children get the care they deserve.

**Disclosures:**

**Ryan H. Rochat, MD, PhD, MS**, Merck Sharpe Dohme: Grant/Research Support

